# UDBRNet: A novel uncertainty driven boundary refined network for organ at risk segmentation

**DOI:** 10.1371/journal.pone.0304771

**Published:** 2024-06-17

**Authors:** Riad Hassan, M. Rubaiyat Hossain Mondal, Sheikh Iqbal Ahamed

**Affiliations:** 1 Institute of Information and Communication Technology, Bangladesh University of Engineering and Technology, Palashi, Dhaka, Bangladesh; 2 Department of Computer Science, Marquette University, Milwaukee, Wisconsin, United States of America; Soochow University, CHINA

## Abstract

Organ segmentation has become a preliminary task for computer-aided intervention, diagnosis, radiation therapy, and critical robotic surgery. Automatic organ segmentation from medical images is a challenging task due to the inconsistent shape and size of different organs. Besides this, low contrast at the edges of organs due to similar types of tissue confuses the network’s ability to segment the contour of organs properly. In this paper, we propose a novel convolution neural network based uncertainty-driven boundary-refined segmentation network (UDBRNet) that segments the organs from CT images. The CT images are segmented first and produce multiple segmentation masks from multi-line segmentation decoder. Uncertain regions are identified from multiple masks and the boundaries of the organs are refined based on uncertainty data. Our method achieves remarkable performance, boasting dice accuracies of 0.80, 0.95, 0.92, and 0.94 for Esophagus, Heart, Trachea, and Aorta respectively on the SegThor dataset, and 0.71, 0.89, 0.85, 0.97, and 0.97 for Esophagus, Spinal Cord, Heart, Left-Lung, and Right-Lung respectively on the LCTSC dataset. These results demonstrate the superiority of our uncertainty-driven boundary refinement technique over state-of-the-art segmentation networks such as UNet, Attention UNet, FC-denseNet, BASNet, UNet++, R2UNet, TransUNet, and DS-TransUNet. UDBRNet presents a promising network for more precise organ segmentation, particularly in challenging, uncertain conditions. The source code of our proposed method will be available at https://github.com/riadhassan/UDBRNet.

## 1 Introduction

Robotic surgery, computer aided diagnosis, targeted radiation therapy require meticulous segmentation of affected organ from adjacent organs [1–5]. The authors of [6] examined the evolution of automatic multi-organ segmentation techniques, comparing traditional methods with deep learning approaches and found that deep learning methods consistently outperformed traditional approaches, indicating their superior efficiency in segmentation tasks. However, despite their success, deep learning models encounter challenges in complex environments [7].

Abdominal organs are difficult to segment due to overlap, inconsistent shape, and uneven size [7–10]. Many convolutional neural network (CNN) based architectures have been proposed to address the challenges posed by diverse organ shapes, sizes, and contrast variations. Among these, DenseNet stands out for its densely interconnected layers, offering a registration-free approach for segmentation tasks [11]. Building upon DenseNet, the authors of [12] further refined the concept with a fully convolutional DenseNet specifically tailored for 2D medical image segmentation.

However, Ronneberger et al. proposed U-Net architecture that has emerged as a popular baseline in medical image segmentation [13]. U-Net’s encoder-decoder design has become a standard framework, inspiring numerous extensions and adaptations to tackle various segmentation challenges. Notable among these extensions are V-Net and 3D U-JAPA-Net, which extend U-Net for volumetric medical image segmentation [14, 15]. Additionally, Yagi et al. [16] developed a UNet based framework tailored for cancer radiotherapy support, with a focus on abdominal organ segmentation.

To enhance U-Net’s performance, researchers have introduced various modifications. Wang et al. contributed Densely Connected Deep U-Net and incorporated densely connected layers to improve abdominal multi-organ segmentation [17, 18]. Oktay et al. integrated attention mechanisms within U-Net architecture, with attention gates at every step of the decoder in Attention UNet [19], and Nazib et al. incorporated uncertainty-based attention within the bottleneck of UNet [20]. Moreover, residual connections and recurrent layers are added with U-Net architecture to feature accumulation in R2UNet [21].

Further advancements include Dense V-Net and improved U-Net architectures which utilize high connectivity between encoder and decoder [22, 23]. Multiple nested U-Net pathways with skip connections have been proposed to capture hierarchical features and context more effectively in UNet++ [24]. Additionally, full-scale skip connections and deep supervision, along with a classification-guided module, have been integrated within U-Net for enhanced medical image segmentation in UNet3+ [25]. Moreover, transformer based network TransUNet [26], DS-TransUNet [27] and EG-TransUNet [28] are proposed to integrate both CNN and transformer based features in medical segmentation. Additionally, boundary aware segmentation networks [29], cascaded spatial shift networks [30], and multiple attention-based segmentation networks [31] have been proposed to address specific challenges in feature refinement. While these networks excel in segmenting relatively consistent and large organs, they may encounter difficulties in smaller, unevenly shaped organs with low contrast around the edges, such as the esophagus and heart [32].

Uncertainty driven organ segmentation improves the performance of medical image segmentation. Recent research has shown that uncertainty levels in convolutional neural networks may reveal segmentation issues. The authors of [33] proposed a segmentation network where they used uncertainty information. To estimate uncertainty, they needed an independent generative adversarial network. The authors of [34] proposed a segmentation network where they needed to input current CT slice, adjacent CT slice, and a prediction mask from another segmentation network to estimate uncertainty. The authors of [35] proposed a segmentation network where multiple manually segmented ground truths were required for every slice of CT image to determine uncertainty.

The conventional network suffers from over segment or under segment around boundary regions due to the similar contrast tissue of adjacent organs and inconsistent organ shape and size. Uncertainty driven deep learning networks either need multiple ground truths or separate independent networks for uncertainty map identification.

To overcome the above-mentioned issues, we have proposed a deep learning based uncertainty driven boundary refined end-to-end network for precise organ segmentation from CT images, UDBRNet, where the organs are segmented, followed by the organs’ boundary refinement with the help of uncertainty information. The CT images are passed through the encoder and the main decoder produces the main mask. Whereas two parallel auxiliary decoders with features drop and random noise layer are used, respectively, for generating two auxiliary masks. Disagreement regions among output masks from multiple decoder lines are considered uncertain regions. Uncertainty information is carried out by utilizing main segmentation masks with uncertainty region data. Both the main segmentation mask and uncertain information are forwarded to the boundary refinement module to refine the boundary residuals of organs. Here, we utilize a hybrid regularizer loss function combining dice and cross-entropy due to considering both shape and entropy penalties during training. We can summarise our contributions in this paper as follows:

- We propose a multi-line decoder-based segmentation module to identify uncertainty regions from single labeled dataset. This consists of one main decoder and two auxiliary decoders, one with noise addition and another with feature drop operation.
- We propose a boundary refinement network that considers uncertainty information along with a segmentation mask to refine the edges of the organs.
- With the segmentation module and the boundary refine network, we propose an end-to-end uncertainty-driven boundary-refined segmentation network, termed UDBRNet, to segment the organs from CT images. We then conduct extensive experiments on two publicly available datasets to compare UDBRNet with eight state-of-the-art segmentation networks.

The remainder of the paper is organized as follows: Section 2 focuses on Methodology; The experimental details are presented in Section 3. Experimental results of our proposed method, UDBRNet, and the existing eight state-of-the-art networks are compared for the two datasets in Section 4; Furthermore, ablation studies for evaluating the effectiveness of different modules of the proposed method are reported in Section 4.3. Finally, the overall conclusion is presented in Section 5.

## 2 Methodology

**Fig 1:**
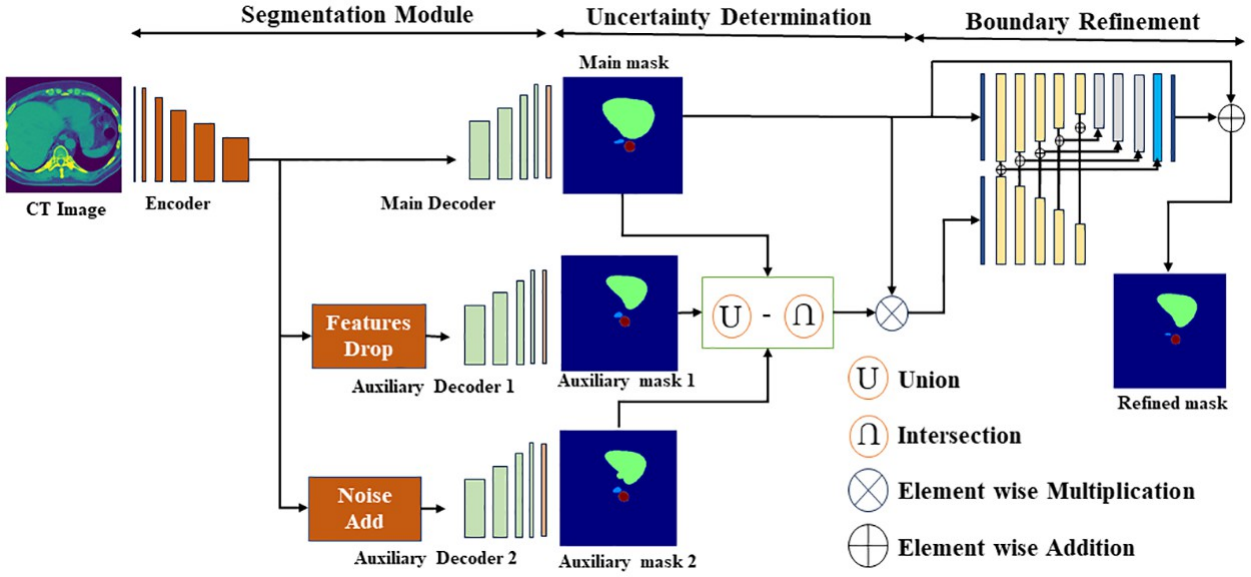
The overall proposed UDBRNet architecture where the segmentation module takes CT image in encoder and generates three segmentation masks from one main and two auxiliary decoders. The encoder’s output is directly fed into the main decoder, while the feature drop operation for one auxiliary decoder and random noise addition operation for another auxiliary decoder are carried out before being supplied. The uncertainty determination module determines uncertainty map based on disagreement among the predicted masks from the multiple decoders. Finally, the boundary refinement module refines each organ’s boundary, considering the uncertainty map and main segmentation mask. The detailed internal network architecture of segmentation, and boundary refinement module are in Fig 2 and uncertainty determination module is in Fig 3.

In our proposed method, we segment organs in three steps. In the first step, organs are segmented from CT images utilizing encoder decoder based architecture where one encoder and multi line decoder are comprised of one main decoder and two auxiliary decoders. Those two auxiliary decoders are incorporated to produce two auxiliary segmentation masks for identifying uncertainty regions. Then the disagreement between union and intersection of all masks is considered as uncertain region. Finally, the segmentation masks’ boundaries are refined by the boundary refinement module with the help of the uncertainty information. The overall architecture of the proposed methods is illustrated in Fig 1.

### 2.1 Segmentation module

**Fig 2:**
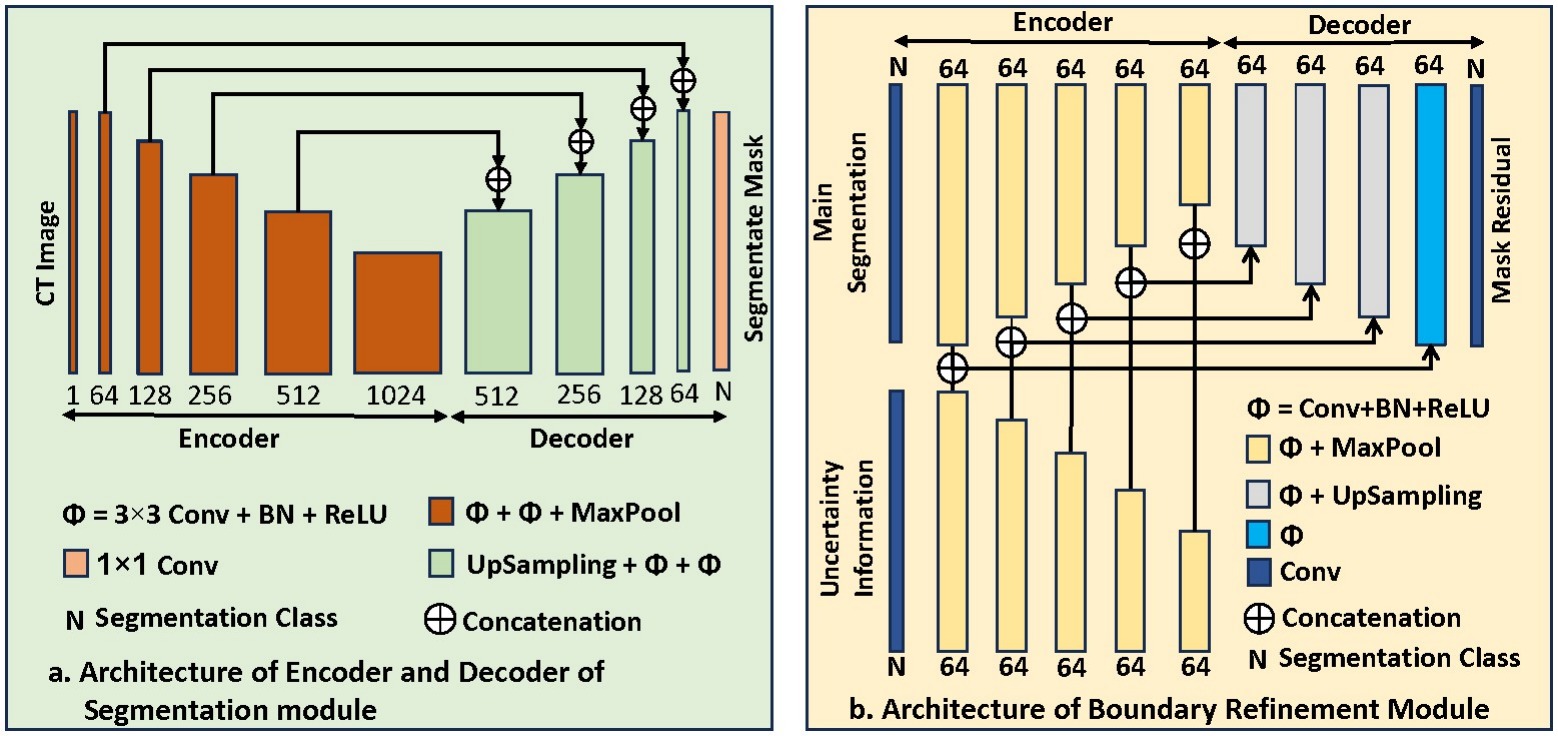
Layer architecture of the proposed method. a) Architecture of Encoder and Decoder of Segmentation Module. One encoder and three decoders (one main and two auxiliaries) are used in UDBRNet’s segmentation module to produce three masks, which are used for uncertainty determination b) Architecture of Boundary Refinement Module, which takes the main segmentation mask and uncertainty information and produces mask residual.

The design of the encoder and decoder of the segmentation module is inspired by the concept of UNet architecture [13]. We implement a block defined in Eq (1) as Φ operation which is comprised of sequential 3 × 3 convolution, Batch Normalization followed by a ReLU activation function. In encoder, after consecutive two Φ operations, 2 × 2 MaxPooling operation is performed. In the first step, we make our single channel CT data into 64 channels and then the number of channels is increased twice in every step compared to the previous step in the encoder. The encoder is represented as *x*^*e*^ in Eq (2). The output from the encoder *x*^*e*^ is directly passed to the main decoder. Additionally, Uniformly Distributed Random Noise (UDRN) and feature drop are employed, respectively, within the encoder and decoder to create two auxiliary decoder lines, which are presented in Eq (3). The output from the encoder is fed to the corresponding decoder. In the decoders, upsampling and then two consecutive Φ operations are performed in every step. In this case, the number of channels in each step becomes half that in the previous step as the reverse of the encoder. In every decoder step, skip connections are added from the corresponding encoder step to retain spatial details, enhance gradient flow, and capture contextual information. Then, 1 × 1 convolution is performed and produces *N* number of channel output in the last step of the decoder, where *N* is the number of segmentation classes. The decoder module is presented in Eq (4). The pictorial depiction of the segmentation module encoder and decoder is in Fig 2(a). After SoftMax operation of output from one main and two auxiliary decoder lines as Eqs (5), (6) and (7), one main *Mask*_*main*_ and two auxiliary segmentation masks *Mask*_*aux*1_ and *Mask*_*aux*2_ are produced respectively in the output of the segmentation module.

$$\begin{array}{c} \Phi \left(\cdot \right)=ReLU(BN\left(Conv_{3\times 3}\left(\cdot \right)\right)) \end{array}$$ (1)

$$\begin{array}{c} x_{i}^{e}=\{\begin{array}{cc} \Phi (\Phi (x)),\; & i=1\\ MaxPool(\Phi \left(\Phi \left(x_{i-1}^{e}\right)\right)),\; & i=2,3,4,5 \end{array} \end{array}$$ (2)

$$\begin{array}{c} x^{b}\left(Dec\right)=\{\begin{array}{cc} x_{5}^{e},\; & Dec=Main\\ FeatureDrop(x_{5}^{e}),\; & Dec=Aux_{1}\\ Noise(x_{5}^{e}),\; & Dec=Aux_{2} \end{array} \end{array}$$ (3)

$$\begin{array}{c} x_{i}^{d}=\{\begin{array}{cc} \Phi (\Phi \left(UpSample\left(x^{b}\left(Dec\right)\right)⊕x_{5-i}^{e}\right)),\; & i=1\\ \Phi (\Phi \left(UpSample\left(x_{i-1}^{d}\right)⊕x_{5-i}^{e}\right)),\; & i=2,3,4\\ Conv_{1\times 1}\left(x_{i-1}^{d}\right)\; & i=5 \end{array} \end{array}$$ (4)

Here, the subscript *i* represents the layer number of the encoders and the decoder.

$$\begin{array}{c} Mask_{main}=SoftMax\left(x_{5}^{d}\right),\;Dec=Main \end{array}$$ (5)

$$\begin{array}{c} Mask_{aux1}=SoftMax\left(x_{5}^{d}\right),\;Dec=Aux_{1} \end{array}$$ (6)

$$\begin{array}{c} Mask_{aux2}=SoftMax\left(x_{5}^{d}\right),\;Dec=Aux_{2} \end{array}$$ (7)

### 2.2 Uncertainty determination module

**Fig 3:**
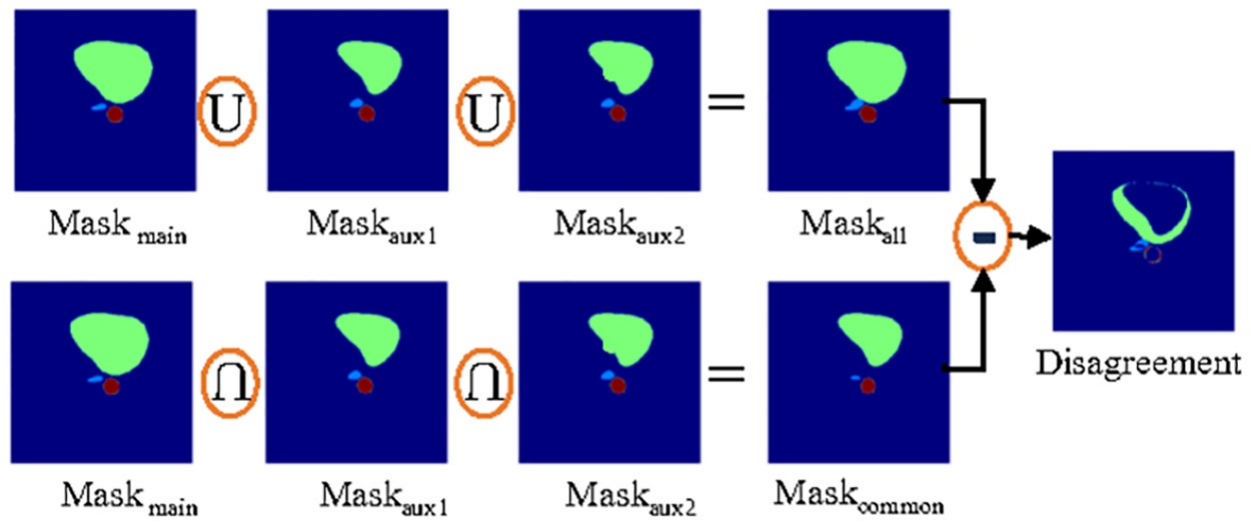
Disagreement region determination from multiple segmentation masks which is produced from one main and two auxiliary decoders of segmentation module.

To identify the uncertainty, firstly, a region is considered as uncertain for a particular organ if any one of the three output masks disagrees with other masks. To carry out the *Disagreement* region like Eq (10), the difference between union and intersection of all three output masks are considered where union represents both agreement and disagreement which is symbolized as *Mask*_*all*_ in Eq (8) and intersection represents only agreement which is symbolized as *Mask*_*common*_ in Eq (9). The process is depicted in Fig 3. Finally, to get *Uncertainty*, the *Mask*_*main*_ is element wise multiplied with *Disagreement* region as Eq (11).

$$\begin{array}{c} Mask_{all}=Mask_{main}\cup Mask_{aux1}\cup Mask_{aux2} \end{array}$$ (8)

$$\begin{array}{c} Mask_{common}=Mask_{main}\cap Mask_{aux1}\cap Mask_{aux2} \end{array}$$ (9)

$$\begin{array}{c} Disagreement=Mask_{all}-Mask_{common} \end{array}$$ (10)

$$\begin{array}{c} Uncertainty=Disagreement\times Mask_{main} \end{array}$$ (11)

### 2.3 Boundary refinement module

In the boundary refinement module, the main segmentation mask *Mask*_*main*_ from Eq (5) and uncertainty information *Uncertainty* from Eq (11) are fed and it produces residual, which is element-wise added with the main mask to refine edge for more accurate segmentation. The refinement module is comprised of two identical encoders and one decoder. The encoders are termed as *y*^*e*^, and *z*^*e*^ in Eqs (12) and (13), respectively. The decoder is symbolized as *y*^*d*^ in Eq (14). The main segmentation mask is sent to *y*^*e*^ and uncertainty region information is passed through *z*^*e*^. In each encoder, at first 3 × 3 convolution layer is employed and it produces 64 channel data. After this, in every step of the encoders, sequential Φ and 2 × 2 MaxPolling operations are performed. Between two encoders, output from the encoder, which encodes the main segmentation mask, is passed to the decoder, where skip connections from both encoders are concatenated in the corresponding layers. In every step of the decoder, Φ, and bi-linear upsampling with scaling factor 2 operations are performed. Finally, according to Eq (15) the convolution is employed to get the residual, in which the number of channels is equal to the number of segmentation classes. Now the residual *Mask*_*residual*_ is element wise added with the main segmentation mask *Mask*_*main*_ for more accurate edge segmentation as Eq (16). The architecture of the boundary refinement module is shown in Fig 2(b).

$$\begin{array}{c} y_{i}^{e}=\{\begin{array}{cc} Conv_{3\times 3}\left(Mask_{main}\right),\; & i=1\\ MaxPool(\Phi \left(y_{i-1}^{e}\right)),\; & i=2,3,4,5 \end{array} \end{array}$$ (12)

$$\begin{array}{c} z_{i}^{e}=\{\begin{array}{cc} Conv_{3\times 3}\left(Uncertainty\right),\; & i=1\\ MaxPool(\Phi \left(y_{i-1}^{e}\right)),\; & i=2,3,4,5 \end{array} \end{array}$$ (13)

$$\begin{array}{c} y_{i}^{d}=\{\begin{array}{cc} UpSample(\Phi \left(y_{5-i}^{e}⊕z_{5-i}^{e}\right)), & i=0\\ UpSample(\Phi \left(y_{i-1}^{d}⊕y_{5-i}^{e}⊕z_{5-i}^{e}\right)), & i=1,2,3\\ \Phi (y_{i-1}^{d}) & i=4 \end{array} \end{array}$$ (14)

$$\begin{array}{c} Mask_{residual}=Conv_{3\times 3}\left(y_{4}^{d}\right) \end{array}$$ (15)

Here, the subscript *i* represents the layer number of the encoders and the decoder.

$$\begin{array}{c} Mask_{refined}=Mask_{main}+Mask_{residual} \end{array}$$ (16)

We implement a loss function by combining dice loss and cross-entropy loss for regularization inspired by [36]. The *Mask*_*main*_, *Mask*_*aux*1_, *Mask*_*aux*2_, and *Mask*_*refined*_ are supervised by adding all losses before backpropagation during the training phase like Eq (17). The loss function L(·) is described in Section 3.4.

$$\begin{array}{c} \begin{array}{cc} Loss= & L(Mask_{groundTruth},Mask_{refined})\;+\\ & L(Mask_{groundTruth},Mask_{main})\;+\\ & L(Mask_{groundTruth},Mask_{aux1})\;+\\ & L(Mask_{groundTruth},Mask_{aux2}) \end{array} \end{array}$$ (17)

## 3 Experiment

### 3.1 Datasets

For evaluating our proposed method, we use two publicly available datasets SegThor and LCTSC.

#### 3.1.1 Segthor

There are 40 patients’ CT scans with manual labeling of four organs at risk (i.e. Esophagus, Heart, Trachea, Aorta) are publicly available. The 32 patients’ data were used for training, 8 patients’ data were utilized for testing. In total, it contains 7390 slices of 512 × 512 images [37].

#### 3.1.2 LCTSC

It is CT scan and label dataset of 60 patients that contains five organs (i.e. Esophagus, Spinal cord, Heart, Left Lung, Right Lung) annotation. The 36 patients’ data are for training and left 12 for testing and 12 for validation. In total, it contains 9,593 slices of 512 × 512 images [38].

### 3.2 Preprocessing

We apply an identical pre-processing pipeline for both datasets. A certain level and window size are used to improve the contrast of medical images. In this instance, window size 400 and level 30 are used in every patient’s CT scan to adjust the appearance of the images more visible. Following the contrast enhancement, the region of interest for organ segmentation, which typically represents the human body, is extracted from the overall CT scan image. This phase removes irrelevant information, like the coach of the CT scanner from CT images. Once the human body part has been cropped, the three-dimensional (3D) voxel data are transformed into a series of two-dimensional (2D) images extracting each slice from the axial axis of CT scan. The image slices are resized from 512 × 512 to 256 × 256 so that they fit in the computation memory. Besides this, data are augmented with rotating, cropping, and padding.

### 3.3 Comparing methods

Our proposed model is compared with the popular eight segmentation networks UNet, Attention UNet, FC-denseNet, BASNet, UNet++, R2UNet, TransUNet, and DS-TransUNet.

#### 3.3.1 UNet

It is an encoder-decoder based convolutional neural network architecture widely used in biomedical image segmentation tasks. It uses skip connections to concatenate feature maps from different levels for improved information flow [13] https://github.com/milesial/Pytorch-UNet.

#### 3.3.2 Attention-UNet

In the Attention U-Net (Atten. UNet) architecture, skip connections and decoders generate attention using attention gate. After element-wise summation, a rectified linear unit (ReLU) activation function combines the extracted features [19] https://github.com/ozan-oktay/Attention-Gated-Networks.

#### 3.3.3 FC-DenseNet

In this method, densely connected blocks extract and reuse features where each dense block links numerous layers, boosting information flow. Transition layers set feature map size and numbers as well as skip connections [12] https://github.com/SimJeg/FC-DenseNet.

#### 3.3.4 UNet++

Cascade UNet or UNet++ enhances the skip connections in the U-Net model by incorporating nested and dense skip pathways. By enhancing the skip connection, it extracts more meaningful features from its input data and it leads to better performance in segmentation [24] https://github.com/MrGiovanni/UNetPlusPlus.

#### 3.3.5 BASNet

It is a segmentation architecture that uses convolution, batch normalization, max pooling, ReLU activation, and bilinear upsampling sequentially in encoding and decoding. The backbone network captures multi-level characteristics from the input, while the boundary is refined to improve boundary segmentation [29] https://github.com/xuebinqin/BASNet.

#### 3.3.6 R2UNet

Residual Recurrent U-Net is a medical image segmentation architecture that combines U-Net structure with residual connections and recurrent layers, improving contextual information integration for enhanced segmentation accuracy [21] https://github.com/navamikairanda/R2U-Net.

#### 3.3.7 TransUNet

TransUNet is a hybrid model that combines transformer and CNN architectures, relying on self-attention processes to efficiently gather global image information. This technique improves medical image segmentation tasks by combining the features of both architectures [26] https://github.com/mkara44/transunet_pytorch.

#### 3.3.8 DS-TransUNet

DS-TransUNet integrates dense supervision and self-attention techniques in a single architecture for medical image segmentation problems. The model has robust connections between the encoder and decoder layers to enhance the flow of gradients and the transfer of information [27] https://github.com/TianBaoGe/DS-TransUNet.

### 3.4 Loss function

For regularization, we utilize a hybrid loss function which is comprised of cross entropy loss and dice loss. Both are very popular loss functions in particular segmentation fields and linear addition of these two losses performs better during segmentation [36]. Cross Entropy loss adds a penalty for the pixel-wise prediction, which is represented in Eq (18) whereas dice loss adds a penalty for the degree of mismatch between the predicted region and the ground truth region for a particular class which is presented in Eq (19). After adding the two losses as Eq (20), the backpropagation is performed due to loss optimization.

$$\begin{array}{c} L_{CE}\left(A,B\right)=-\frac{1}{N}\sum_{i=1}^{N}\;\sum_{c=1}^{C}I_{i,c}\left(A,B\right)\;\text{log}\left(B_{i,c}\right) \end{array}$$ (18)

Here, *N* is the number of samples, C is the number of classes, *I*_*i*,*c*_(*A*, *B*) binary indicator (0 or 1) for whether class *c* is the correctly identified for the *i* − *th* sample between ground truth *A*, and Predicted mask*B*, *B*_*i*,*c*_ is the predicted probability that the *i* − *th* sample belongs to class c.

$$\begin{array}{c} L_{Dice}\left(A,B\right)=1-Dice\left(A,B\right) \end{array}$$ (19)

The *Dice*(*A*, *B*) is defined in Eq (21).

$$\begin{array}{c} L\left(A,B\right)=L_{CE}\left(A,B\right)+L_{Dice}\left(A,B\right) \end{array}$$ (20)

### 3.5 Evaluation metrics

To evaluate our proposed method, we use dice coefficient and Hausdorff Distance (HD) in testing for every comparing method. All the evaluations are performed on 3D data, which is generated by stacking 2D prediction masks. Eq (21) represents the dice that is used to evaluate the degree of overlap between two groups. The dice coefficient ranges from 0 to 1, where a higher value indicates a greater overlap or similarity between the predicted and ground truth masks being compared:

$$\begin{array}{c} Dice\left(A,B\right)=\frac{2\times |A\cap B|}{\left|A|+|B\right|} \end{array}$$ (21)

where *A* and *B* represent ground truth and prediction mask, respectively.

HD metric is a highly informative and useful metric as it serves as an indicator of the degree of dissimilarity of segmentation. It expresses dissimilarity between the boundaries of the surface of estimated and ground truth. A lower HD value signifies a higher degree of similarity, indicating better agreement between the predicted and ground truth of the segmentation mask.

### 3.6 Experiment design

Nine independent experiments are conducted based on eight architectures of comparing methods and our proposed method UDBRNet on two datasets (SegThor and LCTSC). For our proposed method, we use ADAM optimizer with 200 training epochs, a learning rate of 0.001, and batch size of 1. For regularization, we utilize a hybrid regularizer loss function by adding both dice loss and cross-entropy loss which is discussed in Section 3.4.

### 3.7 Implementation

The experiment is implemented using PyTorch 2.1.2. All the models’ training and testing are performed in high-performance computing with Intel Xeron 2.40 GHz processor, 64 GB RAM, and Nvidia V100 GPU.

## 4 Result and discussion

**Fig 4:**
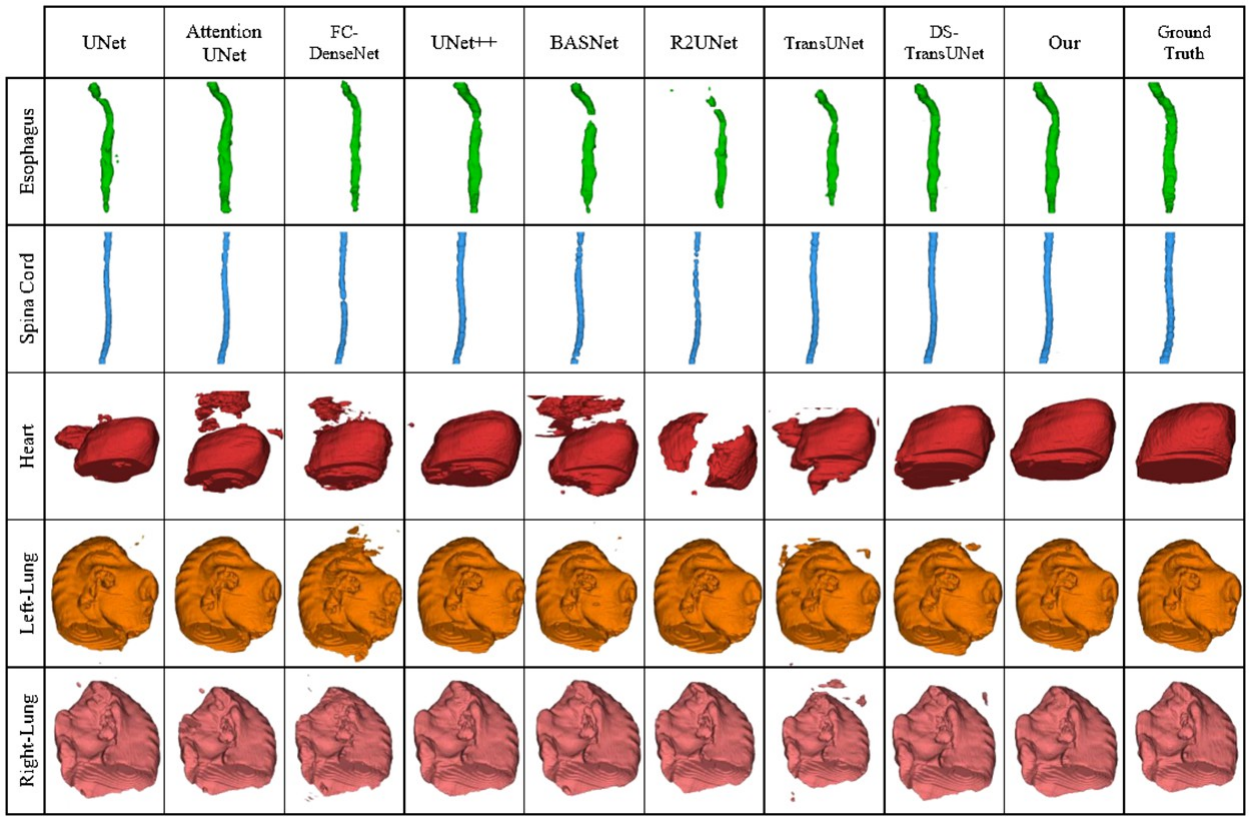
Qualitative results for SegThor dataset using our proposed method UDBRNet and the existing methods.

**Fig 5:**
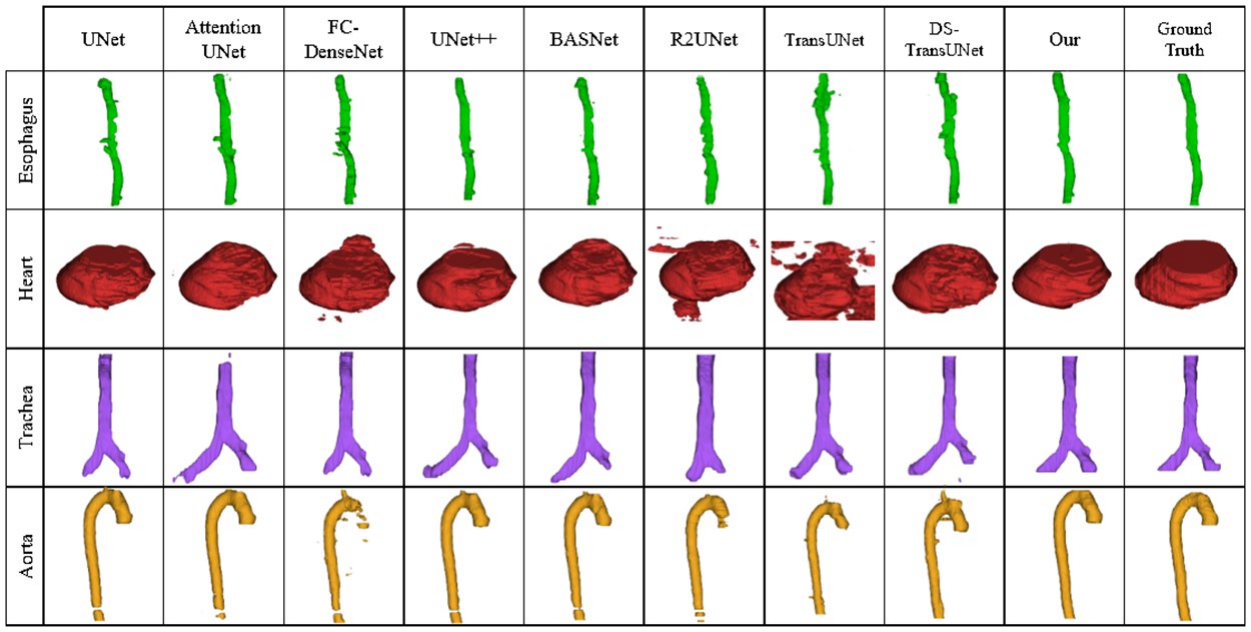
Qualitative results for LCTSC dataset using our proposed method UDBRNet and existing methods.

We conducted a comparative analysis of our proposed method, UDBRNet, against eight state-of-the-art segmentation methods. UDBRNet demonstrated superior performance compared to the other methods. The qualitative results are illustrated in Figs 4 and 5. Beside this, the quantitative results are reported in Tables 1 and 2. In every table, the first row represents the segmentation architecture names, and the rest of the rows represent the organ names, corresponding dice score, and HD value with variance. A model with a higher dice score or a lower HD score is considered to have better performance than other models, which is discussed in Section 3.5. The best performing data for every organ is highlighted with bold text.

**Table 1:** Dice accuracy and HD (± variance) of our proposed method and existing methods for SegThor dataset.

Organ	Metric	UNet	Atten. UNet	FC-DenseNet	UNet++	BASNet	R2UNet	TransUNet	DS-TransUNet	Our
Esophagus	Dice ↑	0.76 ± 0.007	0.75 ± 0.014	0.58 ± 0.027	0.78 ± 0.002	0.76 ± 0.021	0.49 ± 0.011	0.74 ± 0.005	**0.80** ± 0.002	**0.80** ± 0.002
	HD ↓	0.92 ± 0.455	1.03 ± 2.791	1.31 ± 4.764	0.98 ± 0.155	0.90 ± 1.596	1.57 ± 0.583	0.99 ± 2.048	**0.65** ± 0.060	0.81 ± 0.105
Heart	Dice ↑	0.94 ± 0.023	0.89 ± 0.008	0.85 ± 0.027	0.94 ± 0.014	**0.95** ± 0.007	0.80 ± 0.035	0.75 ± 0.067	0.94 ± 0.001	**0.95** ± 0.010
	HD ↓	0.76 ± 9.814	0.92 ± 1.300	1.09 ± 3.709	0.67 ± 1.782	0.68 ± 1.176	2.18 ± 2.348	1.25 ± 14.28	0.69 ± 0.163	**0.64** ± 0.829
Trachea	Dice ↑	0.89 ± 0.011	0.81 ± 0.003	0.78 ± 0.033	0.91 ± 0.005	0.87 ± 0.021	0.84 ± 0.053	0.87 ± 0.023	0.90 ± 0.005	**0.92** ± 0.007
	HD ↓	0.59 ± 1.200	0.53 ± 0.037	0.83 ± 2.173	0.35 ± 0.347	0.61 ± 5.901	1.08 ± 5.047	0.71 ± 8.572	0.35 ± 0.053	**0.33** ± 0.105
Aorta	Dice ↑	0.93 ± 0.002	0.91 ± 0.004	0.83 ± 0.051	**0.94** ± 0.005	0.93 ± 0.007	0.84 ± 0.022	0.89 ± 0.002	0.92 ± 0.000	**0.94** ± 0.000
	HD ↓	0.45 ± 0.312	0.66 ± 2.629	0.90 ± 4.968	**0.35** ± 0.109	0.46 ± 0.254	0.92 ± 10.31	0.63 ± 1.569	0.40 ± 0.161	0.39 ± 0.121

**Table 2:** Dice accuracy and HD (± variance) of our proposed method and existing methods for LCTSC dataset.

Organ	Metric	UNet	Atten. UNet	FC-DenseNet	UNet++	BASNet	R2UNet	TransUNet	DS-TransUNet	Our
Esophagus	Dice ↑	0.50 ± 0.006	0.64 ± 0.011	0.51 ± 0.012	0.68 ± 0.002	0.68 ± 0.007	0.52 ± 0.004	0.63 ± 0.009	0.69 ± 0.007	**0.71** ± 0.001
	HD ↓	3.43 ± 2.378	1.63 ± 8.329	2.29 ± 4.879	1.80 ± 0.603	4.44 ± 7.852	2.13 ± 1.702	2.13 ± 3.301	1.60 ± 0.609	**1.57** ± 0.177
Spinal Cord	Dice ↑	0.86 ± 0.009	0.87 ± 0.010	0.86 ± 0.004	0.88 ± 0.000	0.70 ± 0.023	0.79 ± 0.011	0.88 ± 0.000	0.88 ± 0.000	**0.89** ± 0.000
	HD ↓	0.75 ± 0.146	0.69 ± 0.498	0.68 ± 0.004	0.67 ± 0.006	1.44 ± 2.803	0.72 ± 0.102	0.70 ± 0.006	**0.64** ± 0.001	0.67 ± 0.002
Heart	Dice ↑	0.72 ± 0.042	0.61 ± 0.024	0.78 ± 0.012	0.74 ± 0.043	0.76 ± 0.034	0.45 ± 0.023	0.47 ± 0.017	**0.85** ± 0.001	**0.85** ± 0.035
	HD ↓	4.3 ± 30.015	3.88 ± 9.464	2.00 ± 4.462	2.81 ± 0.634	3.51 ± 4.244	4.12 ± 5.687	10.4 ± 24.16	1.57 ± 0.126	**1.39** ± 0.512
Left-lung	Dice ↑	0.96 ± 0.000	0.96 ± 0.015	0.96 ± 0.000	**0.97** ± 0.000	0.96 ± 0.000	0.94 ± 0.003	0.94 ± 0.005	**0.97** ± 0.000	**0.97** ± 0.000
	HD ↓	0.64 ± 0.008	0.65 ± 2.955	0.67 ± 0.008	0.62 ± 0.046	0.69 ± 0.009	0.65 ± 0.054	0.65 ± 0.244	0.61 ± 0.003	**0.60** ± 0.011
Right-lung	Dice ↑	**0.97** ± 0.000	0.96 ± 0.001	0.96 ± 0.000	**0.97** ± 0.000	**0.97** ± 0.000	0.95 ± 0.001	0.95 ± 0.01	**0.97** ± 0.000	**0.97** ± 0.000
	HD ↓	0.65 ± 0.004	0.71 ± 0.533	0.63 ± 0.003	0.63 ± 0.001	0.63 ± 0.002	0.67 ± 0.005	0.67 ± 0.492	0.61 ± 0.005	**0.60** ± 0.004

### 4.1 Discussion on the results for SegThor dataset

Eight comparing methods and our proposed method’s experimental results on SegThor dataset are presented in Table 1. Our proposed method outperforms existing approaches on the SegThor dataset and achieves 0.80, 0.95, 0.92, 0.94 dice score and 0.81, 0.64, 0.33, 0.39 HD for *esophagus, heart, trachea,* and *aorta*, respectively which demonstrate significant enhancements in segmentation accuracy for different organs. Our approach consistently surpasses baseline models like UNet, Attention UNet, R2UNet, UNet++, FC-DenseNet, BASNet, TransUNet, and DS-TransUNet attaining superior dice scores and reduced Hausdorff distances (HD). Moreover, the HD values acquired using our proposed method are typically lower than those of rival models, indicating superior boundary delineation. The results indicate that our method, which incorporates uncertainty estimation, and boundary refinement, significantly improves the accuracy of segmentation and precision of boundaries.

**Fig 6:**
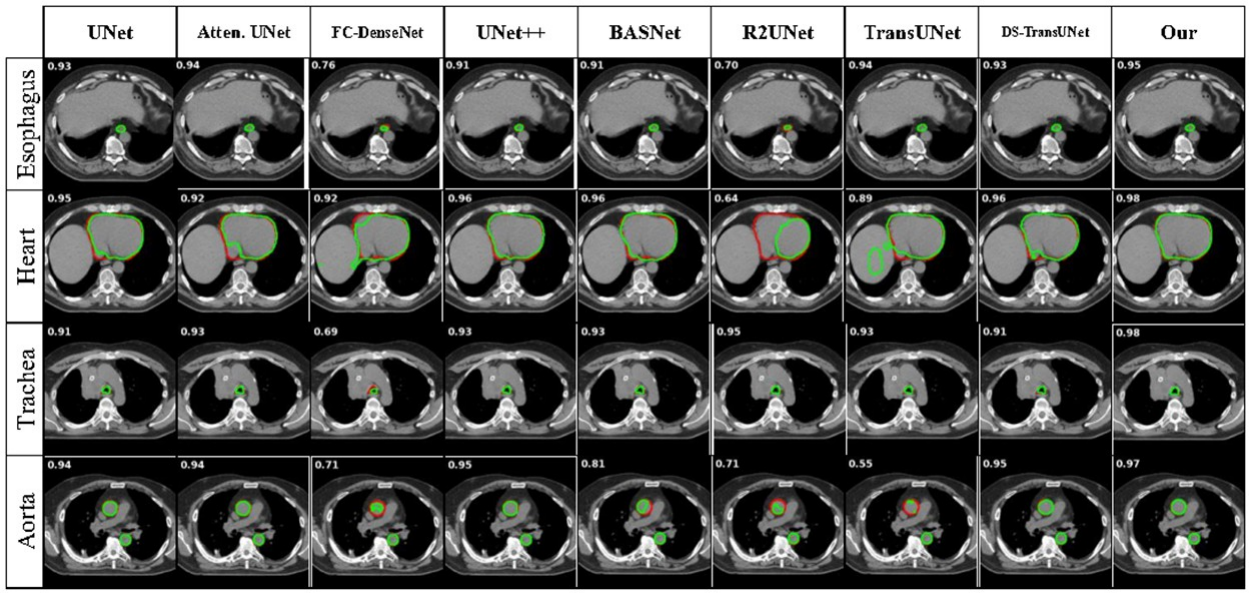
2D contoured segmentation images from SegThor dataset. The red contours depict the accurate representation of the ground truth, while the green contours depict the segmentation achieved by the corresponding architecture. The left-upper corner value on each slice represents the corresponding dice accuracy.

The Figs 4 and 6 present the qualitative outcomes of the 3D and 2D organs illustration for the SegThor dataset respectively. The contouring of the ground truth and predicted results clearly demonstrates that R2Unet, FC-DenseNet under segment, and others over segment in Esophagus segmentation. In Heart segmentation, most of the methods under segment whereas our proposed method more consistently segments Heart. Trachea is under segmented in FC-DenseNet and over segmented in all other comparing methods. In the case of Aorta segmentation R2UNet, BASNet, FC-DenseNet, TransUNet perform under segmentation, and UNet and its successor UNet++ perform over segmentation. Our proposed method outperforms all other methods being compared in terms of segmentation, as we consider uncertainty during boundary refinement, which leads UDBRNet to segment organ boundaries properly and ensures higher accuracy.

### 4.2 Discussion on the results for LCTSC dataset

Table 2 displays the experimental results of all approaches, including our proposed method UDBRNet, on the LCTSC dataset. It achieves dice scores 0.71, 0.89, 0.85, 0.97, 0.97 and HD scores 1.56, 0.67, 1.39, 0.60, 0.60 for *esophagus, spinal cord, heart, left Lung,* and, *right Lung*, respectively which shows substantial improvements in segmentation accuracy compared to existing methods. This indicates that our segmentation quality and border delineation are superior. Significantly, our approach outperforms baseline models such as UNet, Attention UNet, R2UNet, UNet++, FC-DenseNet, BASNet, TransUNet, and DS-TransUNet by a substantial degree, highlighting its efficacy, especially in organs with complex architecture such as the heart and esophagus, highlights the effectiveness of including uncertainty-driven boundary refinement.

**Fig 7:**
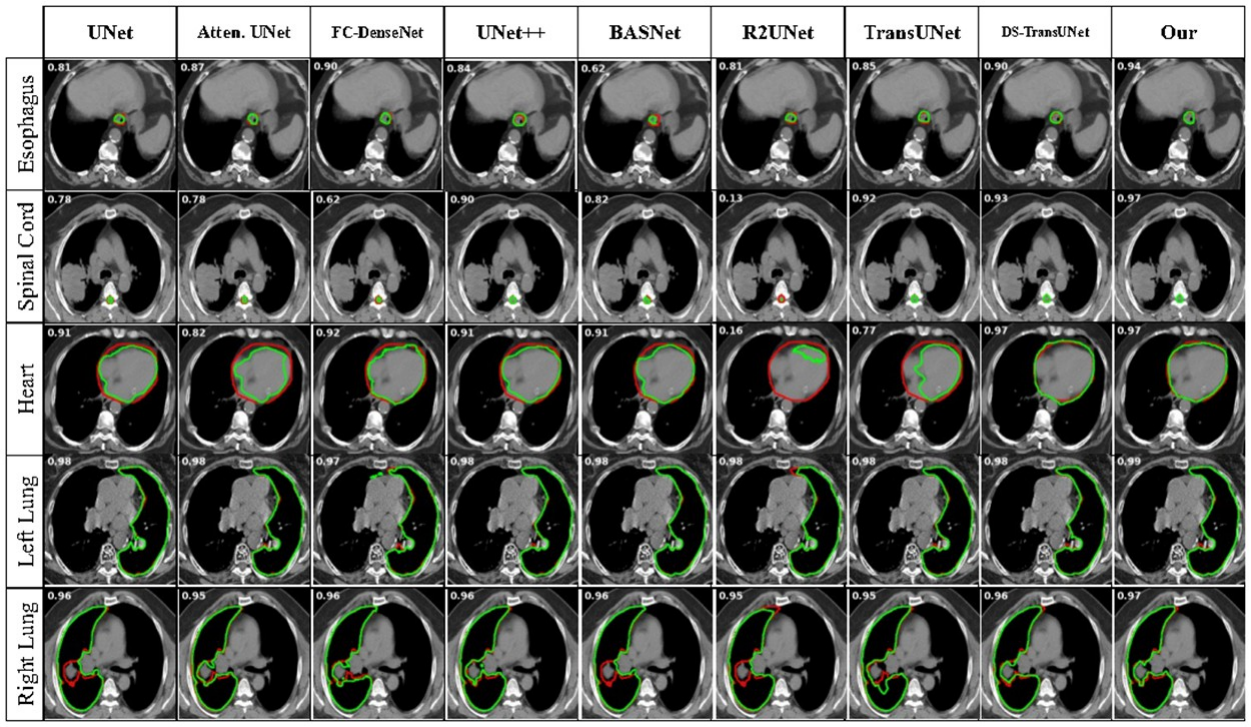
2D contoured segmentation images from LCTSC dataset. The red contours depict the accurate representation of the ground truth, while the green contours depict the segmentation achieved by the corresponding architecture. The left-upper corner value on each slice represents the corresponding dice accuracy.

The qualitative results of the experiments for LCTSC are presented in Figs 5 and 7 which visually reveal that UDBRNet performs better than all other methods in organ segmentation. Though all the segmentation methods show close performance in Left Lung and Right Lung segmentation as the organs contain high contrast tissue around the edges, the comparing methods fail to segment properly when the organs’ shapes are uneven and contain low contrast tissue around the edges like the Esophagus, and Heart. In this unfavorable situation, our proposed method consistently segments organs with a remarkably higher accuracy margin as we consider uncertainty during boundary refinement.

Our suggested segmentation method has been thoroughly evaluated against several state-of-the-art techniques on both the SegThor and LCTSC datasets. While some existing methods like UNet++, DS-TransUNet show competitive performance in terms of dice scores, they often exhibit higher HD values, indicating poorer boundary localization. Whereas, by utilizing uncertainty data in boundary refinement, UDBRNet demonstrates superior ability to reliably delineate organs and consistently outperforms the benchmarked methods across several organs, such as the esophagus, heart, trachea, aorta, spinal cord, left lung, and right lung. Besides this, UDBRNet exposes less variance which indicates the stability of the network and it is necessary for medical applications. The results establish our method as a promising option for organ segmentation from CT images, highlighting its potential to advance the field of medical image analysis and contribute to improved clinical diagnoses and treatment planning. Additional qualitative visualization can be found in S1 Appendix.

### 4.3 Ablation studies

The ablation studies conducted on both the SegThor and LCTSC datasets provided insightful observations regarding the impact of various components within the proposed UDBRNet architecture and reported in Tables 3 and 4 for SegThor and LCTSC dataset, respectively. Only for the segmentation without boundary refinement, we employ the encoder and main decoder from the segmentation module to produce the segmentation mask. Again, for boundary refined segmentation without uncertainty data, we feed the only main segmentation mask from the segmentation module to the boundary refinement module. So, here, the uncertainty determination module and the uncertainty information encoder of the boundary refinement module are not necessary. To check the effectiveness of the auxiliary decoders of segmentation network, every combination of auxiliary decoder 1 and auxiliary decoder 2 are employed during uncertainty calculation. Furthermore, we apply Gaussian Noise (GN) and UDRN separately in our noise addition layer to show the effectiveness of the noise with our proposed network.

**Table 3:** Ablation studies on SegThor dataset.

Module			Auxiliary Decoders			Esophagus		Heart		Trachea		Aorta	
Seg.	UD.	BR.	A.D. 1	A.D. 2	Noise	Dice ↑	HD ↓	Dice ↑	HD ↓	Dice ↑	HD ↓	Dice ↑	HD ↓
√	-	-	-	-	-	0.77 ± 0.002	0.84 ± 0.084	0.93 ± 0.005	0.73 ± 0.504	0.90 ± 0.002	0.34 ± 0.108	0.93 ± 0.001	0.41 ± 0.244
√	-	√	-	-	-	0.78 ± 0.001	0.88 ± 0.096	0.85 ± 0.024	0.84 ± 1.191	0.86 ± 0.021	0.65 ± 3.677	0.92 ± 0.001	0.49 ± 0.204
√	√	√	√	-	-	0.78 ± 0.001	0.83 ± 0.109	0.92 ± 0.007	0.88 ± 0.928	0.91 ± 0.001	0.35 ± 0.190	0.92 ± 0.001	0.48 ± 0.283
√	√	√	-	√	GN	0.75 ± 0.004	0.90 ± 0.552	0.91 ± 0.011	0.88 ± 4.758	0.91 ± 0.004	0.39 ± 0.348	0.91 ± 0.002	0.57 ± 0.649
√	√	√	-	√	UDRN	0.74 ± 0.008	0.88 ± 6.372	0.91 ± 0.028	0.89 ± 12.70	0.90 ± 0.004	0.36 ± 0.936	0.91 ± 0.002	0.49 ± 0.441
√	√	√	√	√	GN	0.76 ± 0.004	**0.79** ± 0.161	0.91 ± 0.011	0.88 ± 4.465	0.91 ± 0.004	0.38 ± 1.195	0.90 ± 0.001	0.53 ± 0.258
√	√	√	√	√	UDRN	**0.80** ± 0.002	0.81 ± 0.105	**0.95** ± 0.010	**0.64** ± 0.829	**0.92** ± 0.007	**0.33** ± 0.105	**0.94** ± 0.000	**0.39** ± 0.121

Note: Seg. = Segmentation, UD. = Uncertainty Determination, BR. = Boundary Refinement, A.D. 1 = Auxiliary Decoder 1 (Feature Drop), A.D. 2 = Auxiliary Decoder 2 (Add Noise)

**Table 4:** Ablation studies on the LCTSC dataset.

Module			Auxiliary Decoders			Esophagus		Spinal Cord		Heart		Lung(L)		Lung(R)	
Seg.	UD.	BR.	A.D. 1	A.D. 2	Noise	Dice ↑	HD ↓	Dice ↑	HD ↓	Dice ↑	HD ↓	Dice ↑	HD ↓	Dice ↑	HD ↓
√	-	-	-	-	-	0.68 ± 0.005	2.47 ± 1.595	0.87 ± 0.000	0.78 ± 0.014	0.81 ± 0.020	2.06 ± 1.179	0.96 ± 0.000	0.65 ± 0.032	**0.97** ± 0.000	0.62 ± 0.006
√	-	√	-	-	-	0.65 ± 0.005	1.71 ± 1.162	0.88 ± 0.000	0.69 ± 0.004	0.57 ± 0.034	4.09 ± 2.125	0.96 ± 0.000	0.62 ± 0.014	0.96 ± 0.000	0.64 ± 0.023
√	√	√	√	-	-	0.69 ± 0.002	1.59 ± 0.601	0.88 ± 0.000	**0.67** ± 0.003	0.83 ± 0.014	1.67 ± 8.230	0.96 ± 0.000	0.63 ± 0.004	0.96 ± 0.000	0.63 ± 0.005
√	√	√	-	√	GN	0.69 ± 0.002	1.84 ± 0.703	0.88 ± 0.000	0.68 ± 0.003	0.82 ± 0.014	1.87 ± 3.639	0.96 ± 0.000	0.65 ± 0.008	**0.97** ± 0.000	0.63 ± 0.009
√	√	√	-	√	UDRN	0.67 ± 0.004	1.60 ± 0.923	0.88 ± 0.000	0.68 ± 0.006	0.81 ± 0.026	1.66 ± 9.251	0.96 ± 0.000	0.64 ± 0.057	**0.97** ± 0.000	0.67 ± 0.002
√	√	√	√	√	GN	0.68 ± 0.004	1.63 ± 1.784	0.88 ± 0.000	0.68 ± 0.008	0.81 ± 0.016	2.06 ± 11.65	0.95 ± 0.001	0.67 ± 0.109	**0.97** ± 0.000	0.66 ± 0.011
√	√	√	√	√	UDRN	**0.71** ± 0.001	**1.57** ± 0.177	**0.89** ± 0.000	**0.67** ± 0.002	**0.85** ± 0.035	**1.39** ± 0.512	**0.97** ± 0.000	**0.60** ± 0.011	**0.97** ± 0.000	**0.60** ± 0.004

Note: Seg. = Segmentation, UD. = Uncertainty Determination, BR. = Boundary Refinement, A.D. 1 = Auxiliary Decoder 1 (Feature Drop), A.D. 2 = Auxiliary Decoder 2 (Add Noise)

The baseline segmentation module exhibited moderate performance, suggesting its ability to provide initial organ segmentation. However, the incorporation of uncertainty determination and boundary refinement modules resulted in substantial enhancements in segmentation accuracy for all organs. This improvement emphasizes the vital importance of uncertainty information in directing the refinement of the edges of organs. The network exhibits similar performance when using a single auxiliary decoder, whether it is a decoder with dropped features or a decoder with added noise, for uncertainty determination. The addition of both auxiliary decoders resulted in additional enhancements in segmentation results which emphasizes the capacity of feature dropout and noise injection within the network to identify uncertain regions more rigorously to improve the resilience of the segmentation process. Moreover, the exploration of several noise types uncovered their effectiveness in organ segmentation with UDBRNet, highlighting the need to choose UDRN. The best design, which includes the integration of segmentation, uncertainty determination, and boundary refinement modules, together with both auxiliary decoders and the type of noise is UDRN, consistently achieved the maximum segmentation accuracy for both datasets.

The proposed segmentation method has the potential to be applied to other application areas where the degree of uncertainty is higher, for instance, anomaly detection in security and surveillance, inspection in robotics, and object segmentation in adverse weather conditions for self-driving cars.

## 5 Conclusion

In this work, we proposed an end-to-end uncertainty driven boundary refined segmentation architecture for medical image segmentation which consists of segmentation, uncertainty determination, and, boundary refined module. The segmentation module produces three output masks from the main and two auxiliary decoder lines. Based on disagreement among the three masks, uncertain regions are identified. Utilizing both the main segmentation mask and the uncertainty information, the boundary refinement module produces the refined segmentation mask. Our proposed method is tested on two publicly available datasets and compared with eight state-of-the-art segmentation architectures. Our method outperforms all others, specifically in organs whose size as well as shape are inconsistent and have low contrast tissue with adjacent organs. Like this, this network has the potential to segment more reliably in uncertain environments. In the future, research may be done to minimize the complexity of the underlying architecture and segment organs more precisely.

## Supporting information

S1 Appendix Contoured image for SegThor and LCTSC dataset. This file contains a comparative visual representation with multiple contoured slices for every organ of SegThor and LCTSC dataset.
(PDF)

## References

[1] Jalalifar SA, Soliman H, Sahgal A, Sadeghi-Naini A. Automatic Assessment of Stereotactic Radiation Therapy Outcome in Brain Metastasis Using Longitudinal Segmentation on Serial MRI. IEEE Journal of Biomedical and Health Informatics. 2023;27(6):2681–2692. doi: 10.1109/JBHI.2023.3235304 37018589

[2] Kolbinger FR, Bodenstedt S, Carstens M, Leger S, Krell S, Rinner FM, et al. Artificial Intelligence for context-aware surgical guidance in complex robot-assisted oncological procedures: An exploratory feasibility study. European Journal of Surgical Oncology. 2023; p. 106996. doi: 10.1016/j.ejso.2023.106996 37591704

[3] Fredriksen V, Sevle SOM, Pedersen A, Langø T, Kiss G, Lindseth F. Teacher-student approach for lung tumor segmentation from mixed-supervised datasets. PLOS ONE. 2022;17(4):1–14. doi: 10.1371/journal.pone.0266147 35381046 PMC8982833

[4] Darapaneni N, Paduri AR, Gulani J, Aithu S, Santhosh MM, Varghese S. Nuclei Segmentation Approach for Computer Aided Diagnosis. In: Multi-disciplinary Trends in Artificial Intelligence. Springer Nature Switzerland; 2023. p. 368–379.

[5] Nishiyama D, Iwasaki H, Taniguchi T, Fukui D, Yamanaka M, Harada T, et al. Deep generative models for automated muscle segmentation in computed tomography scanning. PLOS ONE. 2021;16(9):1–11. doi: 10.1371/journal.pone.0257371 34506602 PMC8432798

[6] Ma J, Zhang Y, Gu S, Zhu C, Ge C, Zhang Y, et al. AbdomenCT-1K: Is Abdominal Organ Segmentation a Solved Problem? IEEE Transactions on Pattern Analysis and Machine Intelligence. 2022;44(10):6695–6714. doi: 10.1109/TPAMI.2021.3100536 34314356

[7] Kaur H, Kaur N, Neeru N. Evolution of multiorgan segmentation techniques from traditional to deep learning in abdominal CT images—A systematic review. Displays. 2022;73:102223. doi: 10.1016/j.displa.2022.102223

[8] Bongratz F, Rickmann AM, Wachinger C. Abdominal organ segmentation via deep diffeomorphic mesh deformations. Scientific Reports. 2023;13(1):18270. doi: 10.1038/s41598-023-45435-2 37880251 PMC10600339

[9] Shen N, Wang Z, Li J, Gao H, Lu W, Hu P, et al. Multi-organ segmentation network for abdominal CT images based on spatial attention and deformable convolution. Expert Systems with Applications. 2023;211:118625. doi: 10.1016/j.eswa.2022.118625

[10] Bilic P, Christ P, Li HB, Vorontsov E, Ben-Cohen A, Kaissis G, et al. The Liver Tumor Segmentation Benchmark (LiTS). Medical Image Analysis. 2023;84:102680. doi: 10.1016/j.media.2022.102680 36481607 PMC10631490

[11] Huang G, Liu Z, Van Der Maaten L, Weinberger KQ. Densely Connected Convolutional Networks. In: 2017 IEEE Conference on Computer Vision and Pattern Recognition (CVPR); 2017. p. 2261–2269.

[12] Jégou S, Drozdzal M, Vazquez D, Romero A, Bengio Y. The One Hundred Layers Tiramisu: Fully Convolutional DenseNets for Semantic Segmentation. In: 2017 IEEE Conference on Computer Vision and Pattern Recognition Workshops (CVPRW); 2017. p. 1175–1183.

[13] Ronneberger O, Fischer P, Brox T. U-Net: Convolutional Networks for Biomedical Image Segmentation. In: Medical Image Computing and Computer-Assisted Intervention—MICCAI 2015. Springer International Publishing; 2015. p. 234–241.

[14] Milletari F, Navab N, Ahmadi SA. V-Net: Fully Convolutional Neural Networks for Volumetric Medical Image Segmentation. arXiv preprint arXiv:160604797. 2016;abs/1606.0:1–11.

[15] Kakeya H, Okada T, Oshiro Y. 3D U-JAPA-Net: Mixture of Convolutional Networks for Abdominal Multi-organ CT Segmentation. In: Medical Image Computing and Computer Assisted Intervention—MICCAI 2018. Springer International Publishing; 2018. p. 426–433.

[16] Yagi N, Nii M, Kobashi S. Abdominal Organ Area Segmentation using U-Net for Cancer Radiotherapy Support. In: 2019 IEEE International Conference on Systems, Man and Cybernetics (SMC); 2019. p. 1210–1214.

[17] Wang ZH, Liu Z, Song YQ, Zhu Y. Densely connected deep U-Net for abdominal multi-organ segmentation. In: 2019 IEEE International Conference on Image Processing (ICIP); 2019. p. 1415–1419.

[18] Wang Y, Zhao L, Wang M, Song Z. Organ at risk segmentation in head and neck ct images using a two-stage segmentation framework based on 3D U-Net. IEEE Access. 2019;7:144591–144602. doi: 10.1109/ACCESS.2019.2944958

[19] Oktay O, Schlemper J, Folgoc LL, Lee M, Heinrich M, Misawa K, et al. Attention U-Net: Learning Where to Look for the Pancreas. In: Medical Imaging with Deep Learning; 2018. Available from: https://openreview.net/forum?id=Skft7cijM.

[20] Nazib A, Hassan R, Islam Z, Fookes C. Uncertainty Driven Bottleneck Attention U-net for Organ at Risk Segmentation; 2024. doi: 10.48550/arXiv.2303.10796

[21] Alom MZ, Yakopcic C, Taha TM, Asari VK. Nuclei Segmentation with Recurrent Residual Convolutional Neural Networks based U-Net (R2U-Net). In: NAECON 2018—IEEE National Aerospace and Electronics Conference; 2018. p. 228–233.

[22] Kausar A, Razzak I, Shapiai I, Alshammari R. An Improved Dense V-Network for Fast and Precise Segmentation of Left Atrium. In: 2021 International Joint Conference on Neural Networks (IJCNN); 2021. p. 1–8.

[23] Gao X, Fang L. Improved U-NET Semantic Segmentation Network. In: 2020 39th Chinese Control Conference (CCC); 2020. p. 7090–7095.

[24] Zhou Z, Siddiquee MMR, Tajbakhsh N, Liang J. UNet++: Redesigning Skip Connections to Exploit Multiscale Features in Image Segmentation. IEEE Transactions on Medical Imaging. 2020;39(6):1856–1867. doi: 10.1109/TMI.2019.2959609 31841402 PMC7357299

[25] Huang H, Lin L, Tong R, Hu H, Zhang Q, Iwamoto Y, et al. UNet 3+: A Full-Scale Connected UNet for Medical Image Segmentation. In: ICASSP 2020—2020 IEEE International Conference on Acoustics, Speech and Signal Processing (ICASSP); 2020. p. 1055–1059.

[26] Chen J, Lu Y, Yu Q, Luo X, Adeli E, Wang Y, et al. TransUNet: Transformers Make Strong Encoders for Medical Image Segmentation. arXiv preprint arXiv:210204306. 2021;.

[27] Lin A, Chen B, Xu J, Zhang Z, Lu G, Zhang D. DS-TransUNet: Dual Swin Transformer U-Net for Medical Image Segmentation. IEEE Transactions on Instrumentation and Measurement. 2022;. doi: 10.1109/TIM.2022.3178991

[28] Pan S, Liu X, Xie N, Chong Y. EG-TransUNet: a transformer-based U-Net with enhanced and guided models for biomedical image segmentation. BMC Bioinformatics. 2023;24(1):85. doi: 10.1186/s12859-023-05196-1 36882688 PMC9989586

[29] Qin X, Zhang Z, Huang C, Gao C, Dehghan M, Jagersand M. BASNet: Boundary-Aware Salient Object Detection. In: 2019 IEEE/CVF Conference on Computer Vision and Pattern Recognition (CVPR); 2019. p. 7471–7481.

[30] Shao Y, Zhou K, Zhang L. CSSNet: Cascaded spatial shift network for multi-organ segmentation. Computers in Biology and Medicine. 2024;170:107955. doi: 10.1016/j.compbiomed.2024.107955 38215618

[31] Li X, Qin X, Huang C, Lu Y, Cheng J, Wang L, et al. SUnet: A multi-organ segmentation network based on multiple attention. Computers in Biology and Medicine. 2023;167:107596. doi: 10.1016/j.compbiomed.2023.107596 37890423

[32] Fu Y, Lei Y, Wang T, Curran WJ, Liu T, Yang X. A review of deep learning based methods for medical image multi-organ segmentation. Physica Medica: European Journal of Medical Physics. 2021;85:107–122. doi: 10.1016/j.ejmp.2021.05.003 33992856 PMC8217246

[33] Hu X, Guo R, Chen J, Li H, Waldmannstetter D, Zhao Y, et al. Coarse-to-Fine Adversarial Networks and Zone-Based Uncertainty Analysis for NK/T-Cell Lymphoma Segmentation in CT/PET Images. IEEE Journal of Biomedical and Health Informatics. 2020;24(9):2599–2608. doi: 10.1109/JBHI.2020.2972694 32054593

[34] Li X, Luo G, Wang W, Wang K, Gao Y, Li S. Hematoma Expansion Context Guided Intracranial Hemorrhage Segmentation and Uncertainty Estimation. IEEE Journal of Biomedical and Health Informatics. 2022;26(3):1140–1151. doi: 10.1109/JBHI.2021.3103850 34375295

[35] Yang H, Shen L, Zhang M, Wang Q. Uncertainty-Guided Lung Nodule Segmentation with Feature-Aware Attention. In: Medical Image Computing and Computer Assisted Intervention—MICCAI 2022. Springer Nature Switzerland; 2022. p. 44–54.

[36] Galdran A, Carneiro G, Ballester MAG. On the Optimal Combination of Cross-Entropy and Soft Dice Losses for Lesion Segmentation with Out-of-Distribution Robustness. In: Diabetic Foot Ulcers Grand Challenge. Springer International Publishing; 2023. p. 40–51.

[37] Lambert Z, Petitjean C, Dubray B, Kuan S. SegTHOR: Segmentation of Thoracic Organs at Risk in CT images. In: 2020 Tenth International Conference on Image Processing Theory, Tools and Applications (IPTA); 2020. p. 1–6. Available from: https://competitions.codalab.org/competitions/21145.

[38] Yang J, Sharp G, Veeraraghavan H, Van Elmpt W, Dekker A, Lustberg T, et al. Data from Lung CT Segmentation Challenge 2017 (LCTSC); 2017. Available from: https://wiki.cancerimagingarchive.net/x/e41yAQ.

