# Supplementary material for: UDBRNet: A novel uncertainty driven boundary refined network for organ at risk segmentation
Source: PLoS One. 2024 Jun 17;19(6):e0304771. doi: 10.1371/journal.pone.0304771 (PMC11182520; doi:10.1371/journal.pone.0304771)
Supplement: S1 Appendix — This file contains a comparative visual representation with multiple contoured slices for every organ of SegThor and LCTSC dataset. (PDF) [file pone.0304771.s001.pdf]

Supporting File

UDBRNet: A Novel Uncertainty Driven Boundary Refined Network for Organ at Risk Segmentation

Riad Hassan[1], M. Rubaiyat Hossain Mondal[1*], Sheikh Iqbal Ahamed[2]

1 Institute of Information and Communication Technology, Bangladesh University of Engineering and Technology, Palashi, Dhaka, Bangladesh
2 Department of Computer Science, Marquette University, Wisconsin Avenue, Milwaukee, Wisconsin, USA

* rubaiyat97@iict.buet.ac.bd (MRHM)

We proposed UDBRNet, an uncertainty driven boundary refined segmentation network that segments organs and refines boundaries utilizing uncertainty region information. The uncertainty information is extracted by its uncertainty determination module. Our proposed method is compared with six state-of-the-art methods (UNet [1], Attention-UNet [2], FC-DenseNet [3], UNet++ [4], BASNet [5], R2UNet [6], TransUNet [7], DS-TransUNet [8]) in two datasets SegThor [9] and LCTSC [10]. The quantitative and qualitative results show that our proposed method outperforms others. As every organ exists in many slices of CT scan data, five slices for every organ are presented in this supplementary material, and one of those is presented in the main paper. The source code of our proposed method will be available at `https://github.com/riadhassan/UDBRNet`.

For SegThor dataset, Figure. 1, Figure 2, Figure 3, and Figure 4 represent Esophagus, Heart, Trachea, and Aorta respectively. Again, for LCTSC dataset, Figure 5, Figure 6, Figure 7, Figure 8, and Figure 9 represent Esophagus, Heart, Left Lung, Right Lung and spinal cord respectively.

The red contours depict the accurate representation of the ground truth, while the green contours depict the segmentation achieved by the corresponding architecture. The left-upper corner value on each image represents the corresponding dice accuracy.

1 Qualitative result: 2D contoured slices from SegThor Dataset

1.1 Esophagus

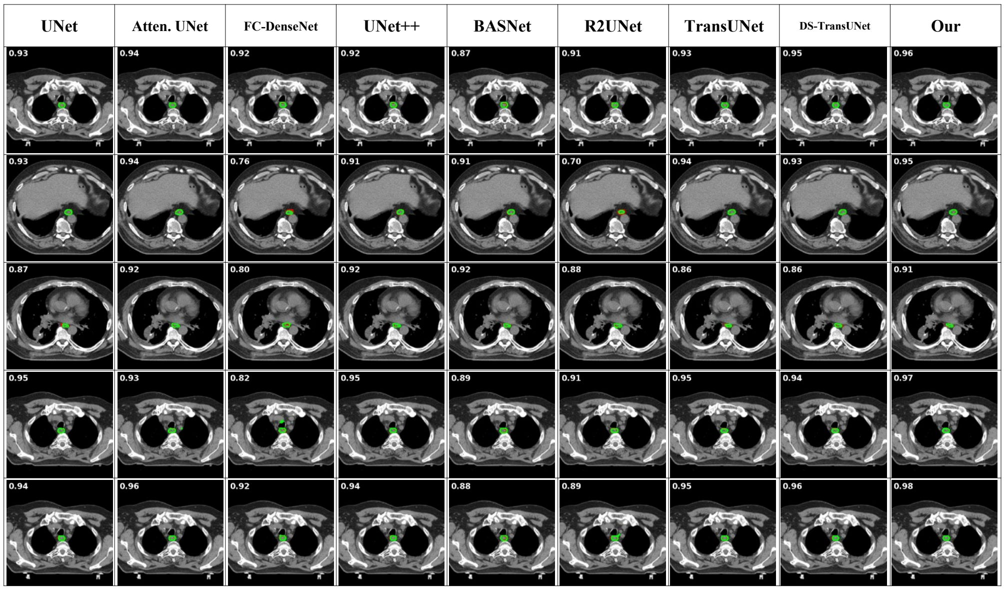

Fig 1. Five slices of organ segmentation from SegThor dataset for the Esophagus representation. The red contours depict the accurate representation of the ground truth, while the green contours depict the segmentation achieved by the corresponding architecture. The left-upper corner value on each slice represents the corresponding dice accuracy.

1.2 Heart

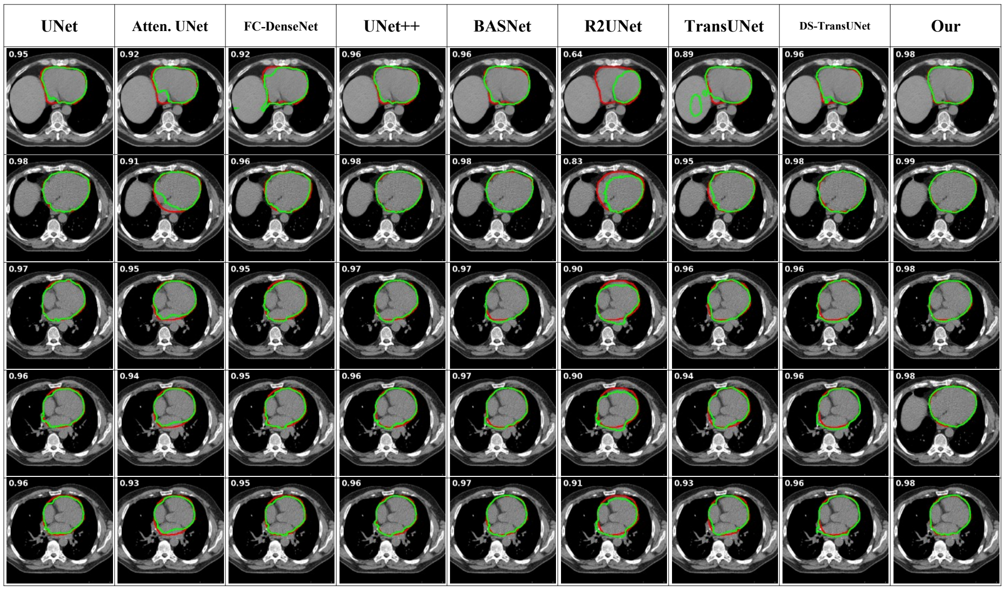

Fig 2. Five slices of organ segmentation from SegThor dataset for the Heart representation. The red contours depict the accurate representation of the ground truth, while the green contours depict the segmentation achieved by the corresponding architecture. The left-upper corner value on each slice represents the corresponding dice accuracy.

1.3 Trachea

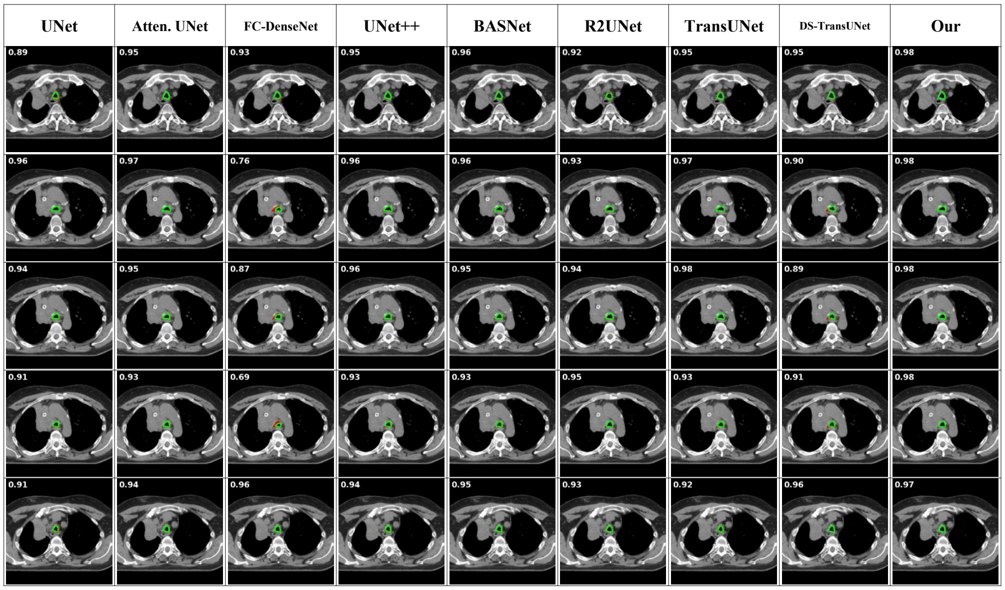

Fig 3. Five slices of organ segmentation from SegThor dataset for the Trachea representation. The red contours depict the accurate representation of the ground truth, while the green contours depict the segmentation achieved by the corresponding architecture. The left-upper corner value on each slice represents the corresponding dice accuracy.

1.4 Aorta

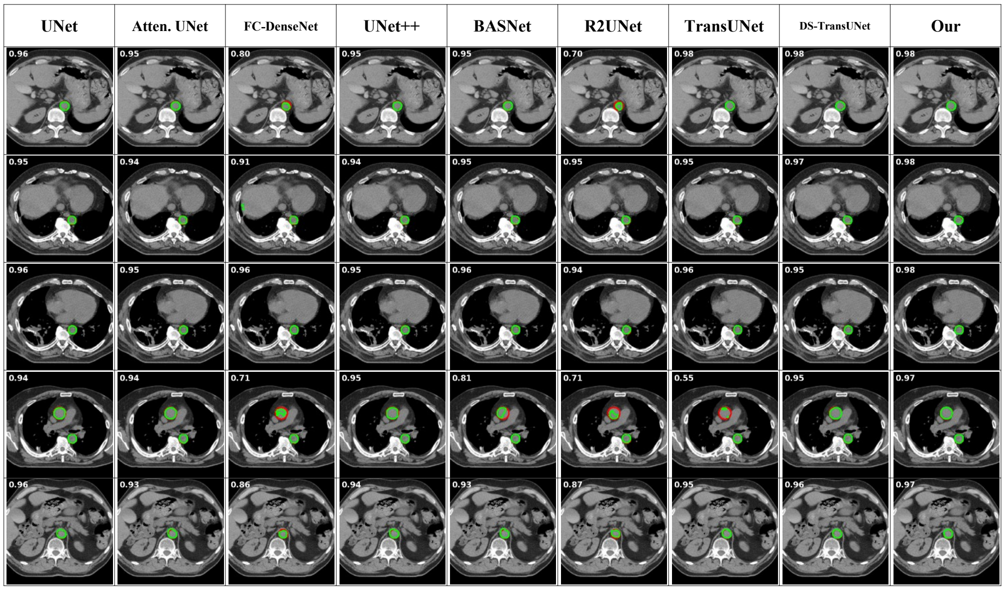

Fig 4. Five slices of organ segmentation from SegThor dataset the Aorta representation. The red contours depict the accurate representation of the ground truth, while the green contours depict the segmentation achieved by the corresponding architecture. The left-upper corner value on each slice represents the corresponding dice accuracy.

2 Qualitative result: 2D contoured slices from LCTSC Dataset

2.1 Esophagus

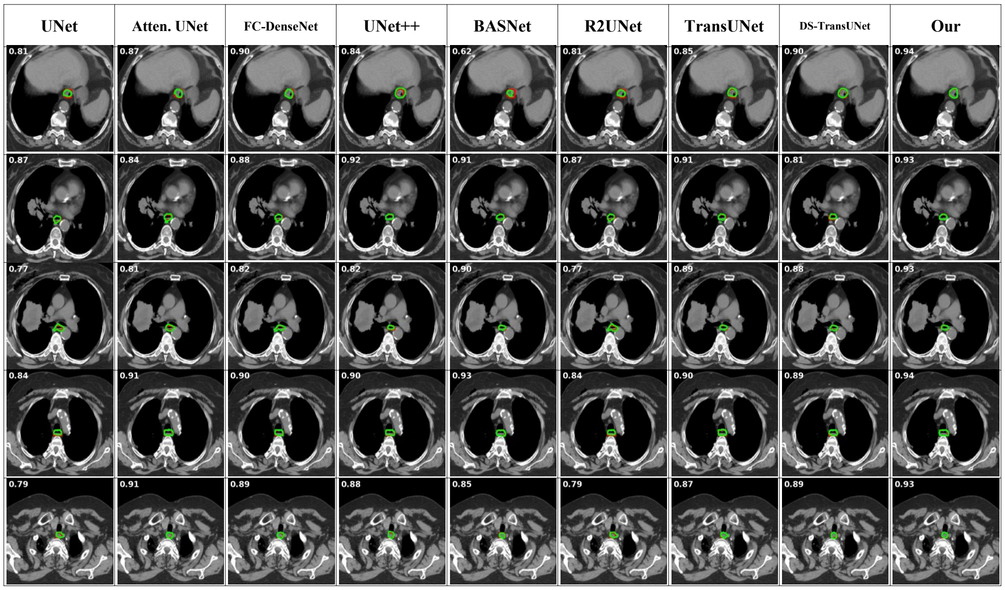

Fig 5. Five slices of organ segmentation from LCTSC dataset for the Esophagus representation. The red contours depict the accurate representation of the ground truth, while the green contours depict the segmentation achieved by the corresponding architecture. The left-upper corner value on each slice represents the corresponding dice accuracy.

2.2 Heart

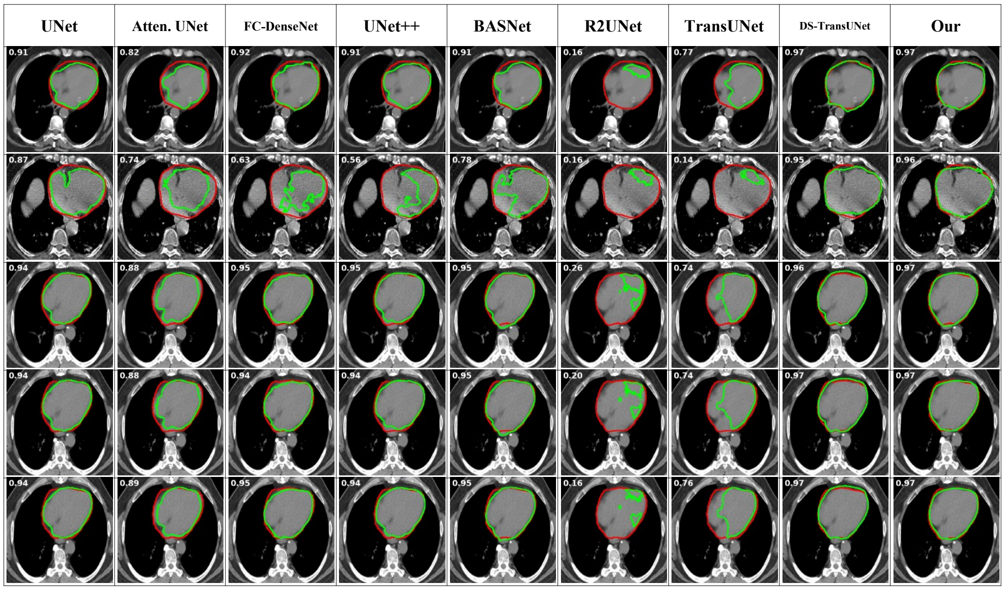

Fig 6. Five slices of organ segmentation from LCTSC dataset for the Heart representation. The red contours depict the accurate representation of the ground truth, while the green contours depict the segmentation achieved by the corresponding architecture. The left-upper corner value on each slice represents the corresponding dice accuracy.

2.3 Lung (Left)

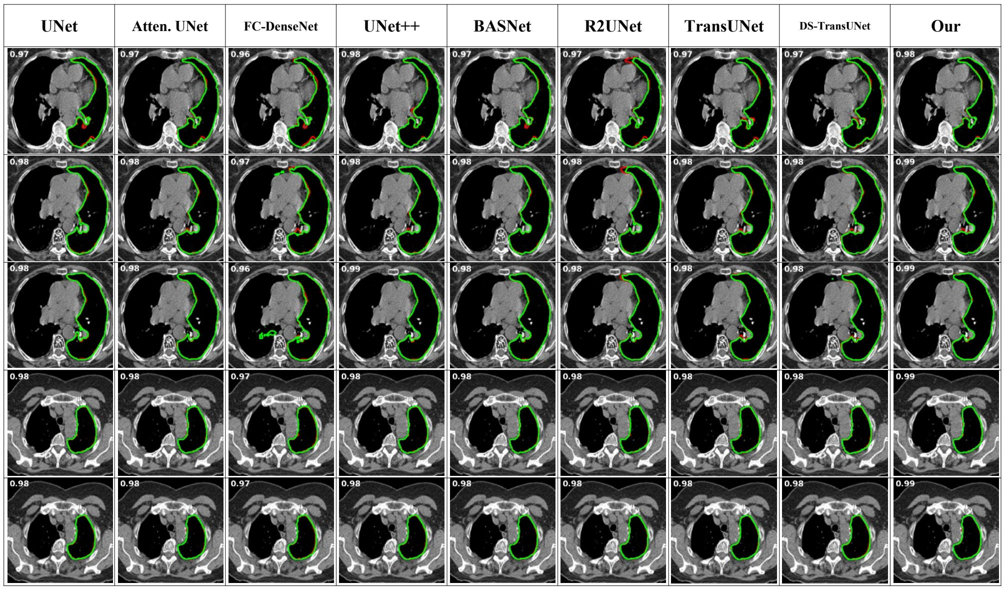

Fig 7. Five slices of organ segmentation from LCTSC dataset of the Lung (Left) representation. The red contours depict the accurate representation of the ground truth, while the green contours depict the segmentation achieved by the corresponding architecture. The left-upper corner value on each slice represents the corresponding dice accuracy.

2.4 Lung (Right)

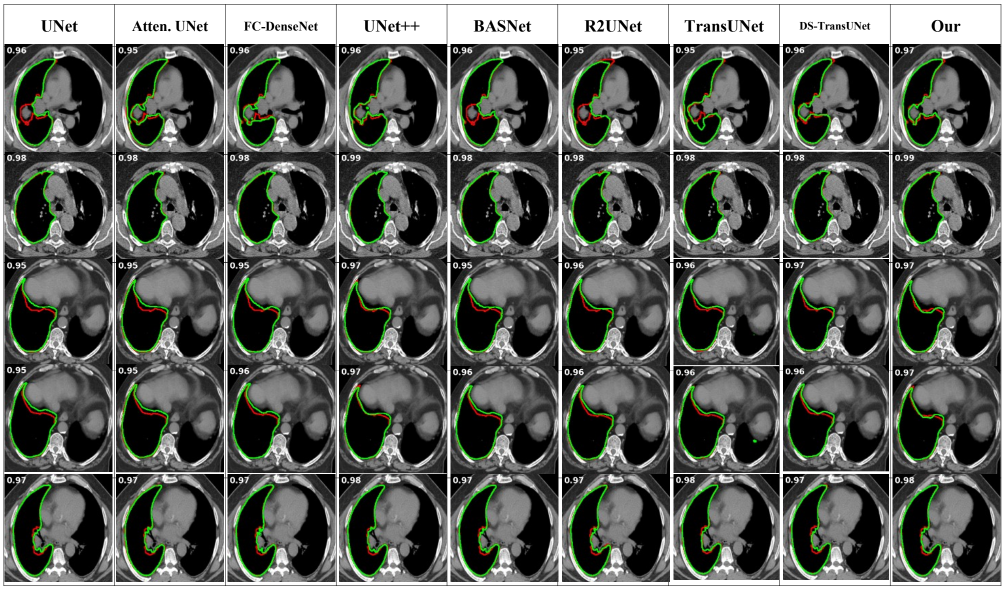

Fig 8. Five slices of organ segmentation from LCTSC dataset for the Lung (Right) representation. The red contours depict the accurate representation of the ground truth, while the green contours depict the segmentation achieved by the corresponding architecture. The left-upper corner value on each slice represents the corresponding dice accuracy.

2.5 Spinal Cord

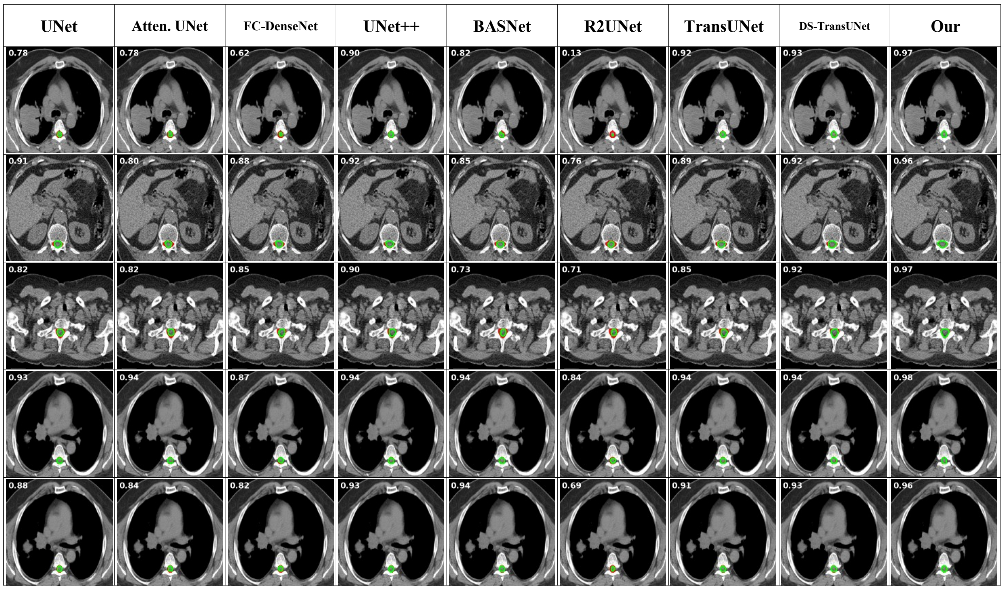

Fig 9. Five slices of organ segmentation from LCTSC dataset for the Spinal Cord representation. The red contours depict the accurate representation of the ground truth, while the green contours depict the segmentation achieved by the corresponding architecture. The left-upper corner value on each slice represents the corresponding dice accuracy.

References

1. Ronneberger O, Fischer P, Brox T. U-Net: Convolutional Networks for Biomedical Image Segmentation. In: Medical Image Computing and Computer-Assisted Intervention – MICCAI 2015. Springer International Publishing; 2015. p. 234–241.

2. Oktay O, Schlemper J, Folgoc LL, Lee M, Heinrich M, Misawa K, et al. Attention U-Net: Learning Where to Look for the Pancreas. In: Medical Imaging with Deep Learning; 2018.Available from: `https://openreview.net/forum?id=Skft7cijM`.

3. Jégou S, Drozdzal M, Vazquez D, Romero A, Bengio Y. The One Hundred Layers Tiramisu: Fully Convolutional DenseNets for Semantic Segmentation. In: 2017 IEEE Conference on Computer Vision and Pattern Recognition Workshops (CVPRW); 2017. p. 1175–1183.

4. Zhou Z, Siddiquee MMR, Tajbakhsh N, Liang J. UNet++: Redesigning Skip Connections to Exploit Multiscale Features in Image Segmentation. IEEE Transactions on Medical Imaging. 2020;39(6):1856–1867. doi:10.1109/TMI.2019.2959609.

5. Qin X, Zhang Z, Huang C, Gao C, Dehghan M, Jagersand M. BASNet: Boundary-Aware Salient Object Detection. In: 2019 IEEE/CVF Conference on Computer Vision and Pattern Recognition (CVPR); 2019. p. 7471–7481.

6. Alom MZ, Yakopcic C, Taha TM, Asari VK. Nuclei Segmentation with Recurrent Residual Convolutional Neural Networks based U-Net (R2U-Net). In: NAECON 2018 - IEEE National Aerospace and Electronics Conference; 2018. p. 228–233.

7. Chen J, Lu Y, Yu Q, Luo X, Adeli E, Wang Y, et al. TransUNet: Transformers Make Strong Encoders for Medical Image Segmentation. arXiv preprint arXiv:210204306. 2021;.

8. Lin A, Chen B, Xu J, Zhang Z, Lu G, Zhang D. DS-TransUNet: Dual Swin Transformer U-Net for Medical Image Segmentation. IEEE Transactions on Instrumentation and Measurement. 2022;.

9. Lambert Z, Petitjean C, Dubray B, Kuan S. SegTHOR: Segmentation of Thoracic Organs at Risk in CT images. In: 2020 Tenth International Conference on Image Processing Theory, Tools and Applications (IPTA); 2020. p. 1–6. Available from: `https://competitions.codalab.org/competitions/21145`.

10. Yang J, Sharp G, Veeraraghavan H, Van Elmpt W, Dekker A, Lustberg T, et al.. Data from Lung CT Segmentation Challenge 2017 (LCTSC); 2017. Available from: `https://wiki.cancerimagingarchive.net/x/e41yAQ`.
